# An outcome model for human bladder cancer: A comprehensive study based on weighted gene co‐expression network analysis

**DOI:** 10.1111/jcmm.14918

**Published:** 2019-12-28

**Authors:** Yaoyi Xiong, Lushun Yuan, Jing Xiong, Huimin Xu, Yongwen Luo, Gang Wang, Lingao Ju, Yu Xiao, Xinghuan Wang

**Affiliations:** ^1^ Department of Urology Zhongnan Hospital of Wuhan University Wuhan China; ^2^ Division of Nephrology Department of Internal Medicine Leiden University Medical Center Leiden The Netherlands; ^3^ Department of Urology Chongqing General Hospital University of Chinese Academy of Sciences Chongqing China; ^4^ Department of Biological Repositories Zhongnan Hospital of Wuhan University Wuhan China; ^5^ Human Genetics Resource Preservation Center of Hubei Province Wuhan China; ^6^ Laboratory of Precision Medicine Zhongnan Hospital of Wuhan University Wuhan China; ^7^ Medical Research Institute Wuhan University Wuhan China

**Keywords:** bladder cancer, Gene Expression Omnibus (GEO), LASSO, prognosis, WGCNA

## Abstract

The precision evaluation of prognosis is crucial for clinical treatment decision of bladder cancer (BCa). Therefore, establishing an effective prognostic model for BCa has significant clinical implications. We performed WGCNA and DEG screening to initially identify the candidate genes. The candidate genes were applied to construct a LASSO Cox regression analysis model. The effectiveness and accuracy of the prognostic model were tested by internal/external validation and pan‐cancer validation and time‐dependent ROC. Additionally, a nomogram based on the parameter selected from univariate and multivariate cox regression analysis was constructed. Eight genes were eventually screened out as progression‐related differentially expressed candidates in BCa. LASSO Cox regression analysis identified 3 genes to build up the outcome model in E‐MTAB‐4321 and the outcome model had good performance in predicting patient progress free survival of BCa patients in discovery and test set. Subsequently, another three datasets also have a good predictive value for BCa patients' OS and DFS. Time‐dependent ROC indicated an ideal predictive accuracy of the outcome model. Meanwhile, the nomogram showed a good performance and clinical utility. In addition, the prognostic model also exhibits good performance in pan‐cancer patients. Our outcome model was the first prognosis model for human bladder cancer progression prediction via integrative bioinformatics analysis, which may aid in clinical decision‐making.

## 1 INTRODUCTION

Bladder cancer (BCa) is the most common malignancy in urinary system. In 2017, the number of deaths caused by BCa has reached 16 870 in US.^1^ Approximately 75% of BCa are diagnosed with non‐muscle‐invasive BCa (NMIBC).^2^ NMIBC patients can achieve better clinical therapeutic effect after transurethral resection combined with anti‐tumour drug intravesical instillation. Unfortunately, there are almost 20% NMIBC patients will progress to muscle‐invasive BCa (MIBC), which still has a poor outcome after systemic therapy.^3^

The evaluation of prognosis is crucial for clinical treatment decision of BCa.^4^ Currently, most of physician's prognostic prediction and treatment decision of BCa are based on American Joint Committee on Cancer (AJCC) TNM staging system.^5^ Due to the heterogeneity of BCa, the prognosis of BCa patients with same stage has significant differences, causing the sensitivity and accuracy of TNM staging system is not as good as expected. Consequently, many researchers are trying to found some certain gene signatures for predicting the prognosis of BCa, such as P53, KI67 and FGFR3.^6^, ^7^, ^8^ However, there is still a huge distance between clinical application and these gene signatures because of lacking enough validation. Therefore, establishing an effective prognostic model for BCa has significant clinical implications.

Recently, many researchers focus on the exploration of microarray data.^9^, ^10^ The common characteristic of survival analysis modelling using microarray data is overfitting. Compared with the cox risk regression analysis, this imperfection can be perfectly solved by the least absolute shrinkage and selection operator (LASSO) cox method.

In the seven datasets of BCa, Weighted gene correlation network analysis (WGCNA) and differentially expressed genes (DEGs) screening was applied to exploring the candidate genes. Based on the candidate genes, the prognostic model was constructed by LASSO cox regression in the discovery dataset of BCa. Then, we validate the performance and accuracy of the prognostic model by several test sets and time‐dependent ROC respectively. Subsequently, univariate and multivariate cox regression analysis were performed and the nomogram was established to validate the clinical utility of the prognostic model. In addition, the performance of the model was also validated in pan‐cancer in TCGA data. Taken together, these results show the three gene signatures can be applied as new independent prognostic biomarkers for predicting the survival of BCa and pan‐cancer patients.

## 2 MATERIAL AND METHODS

### 2.1 Workflow, clinical samples and data acquisition

**Figure 1:**
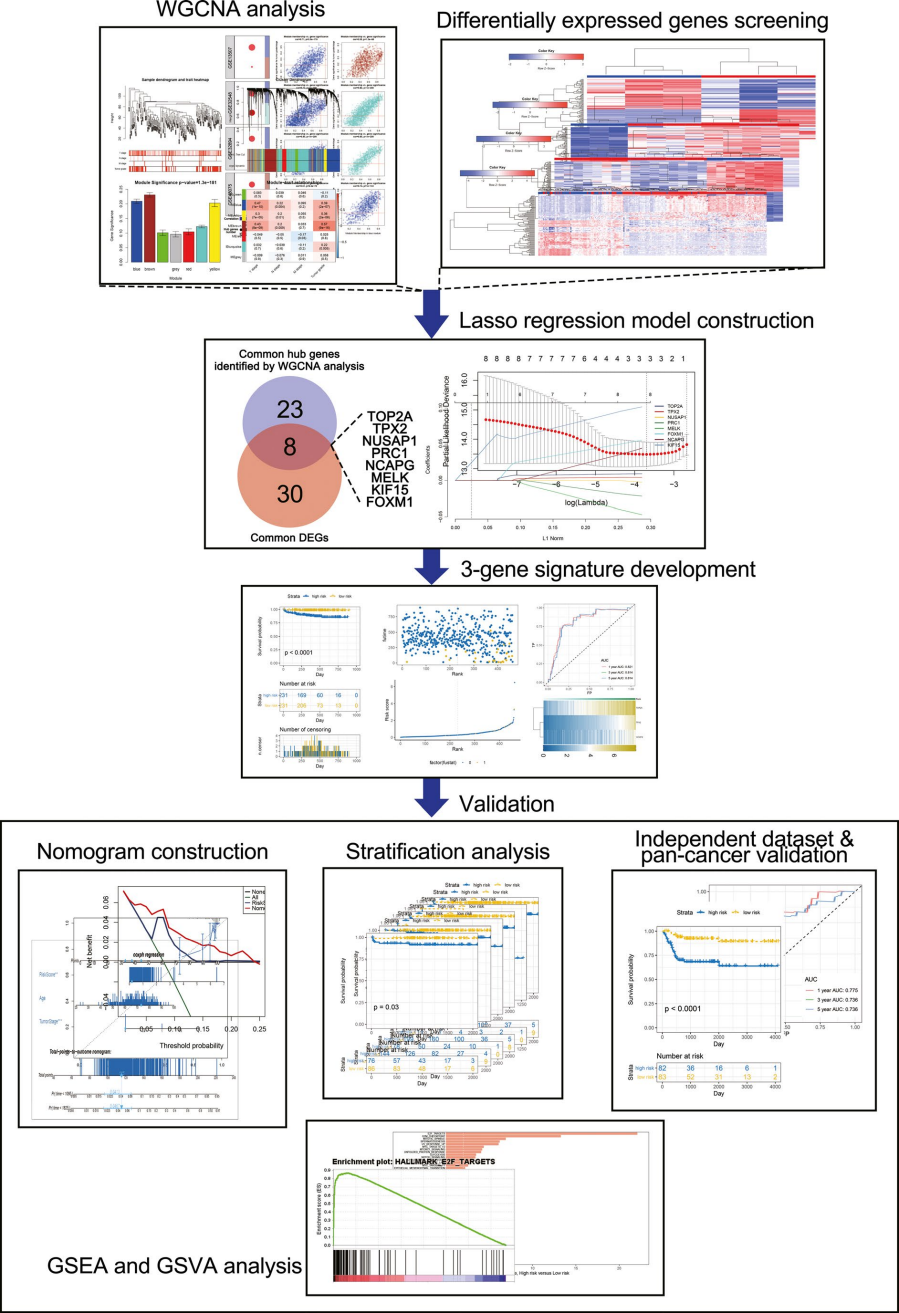
The flow chart of this study

The flow chart was showed in Figure 1. Transcriptome gene expression profiles of bladder cancer and corresponding clinical information of patients were obtained from Gene Expression Omnibus (GEO) database (http://www.ncbi.nlm.nih.gov/geo/) and ArrayExpress database (https://www.ebi.ac.uk/arrayexpress/). Meanwhile, our group established a microarray analysis using mRNA isolated from the three stage II bladder cancer tissues and the three normal bladder tissues, as described by Wang et al^11^ in 2016 (http://www.ncbi.nlm.nih.gov/geo/query/acc.cgi?acc=GSE76211). Detailed information for the 9 independent datasets including E‐MTAB‐4321, http://www.ncbi.nlm.nih.gov/geo/query/acc.cgi?acc=GSE13507, http://www.ncbi.nlm.nih.gov/geo/query/acc.cgi?acc=GSE32548, http://www.ncbi.nlm.nih.gov/geo/query/acc.cgi?acc=GSE32894, http://www.ncbi.nlm.nih.gov/geo/query/acc.cgi?acc=GSE40355, http://www.ncbi.nlm.nih.gov/geo/query/acc.cgi?acc=GSE48075, http://www.ncbi.nlm.nih.gov/geo/query/acc.cgi?acc=GSE5287, http://www.ncbi.nlm.nih.gov/geo/query/acc.cgi?acc=GSE7476 and http://www.ncbi.nlm.nih.gov/geo/query/acc.cgi?acc=GSE76211 were listed in Table S1.

### 2.2 Weighted gene co‐expression network construction and progression‐related genes identification

For WGCNA analysis, we first calculated the standard deviation values for gene expression in each dataset, ranked by it and chose the top 25% for the following analysis.^12^ Using ‘WGCNA’ R package, we first deleted the outliers in each dataset.^13^ Then, to ensure a scale‐free network, proper soft‐thresholding parameter β was chosen and genes with similar expression pattern were clustered into the same module. Combined the modules with the clinical information, we could finally identify the key genes related to tumour progression. And common identified key genes in 4 datasets were considered as candidates playing roles in BCa progression.

### 2.3 Data processing and differentially expressed genes (DEGs) screening

RMA background correction for the raw expression data was first applied for the DEGs screening. Meanwhile, we performed log2 transformation and normalization for processed signals. Then, ‘affy’ R package was applied to summarize the median‐polish probe sets and the Affymetrix annotation files were used for probe annotation. Before the mRNA sequencing analyses, mRNA‐seq data was first normalized by ‘limma’ package and the expression value of each mRNA for each sample were normalized to generate the expression matrix. The normalized data were arranged in the order of tumour tissues and paracancerous normal tissues. In addition, the DEGs were screened by making comparisons between tumour tissues and paracancerous normal tissues. |log2FC| > 1 and FDR < 0.05 were used as the cut‐off threshold for datasets http://www.ncbi.nlm.nih.gov/geo/query/acc.cgi?acc=GSE13507, http://www.ncbi.nlm.nih.gov/geo/query/acc.cgi?acc=GSE40355 and http://www.ncbi.nlm.nih.gov/geo/query/acc.cgi?acc=GSE7476; for http://www.ncbi.nlm.nih.gov/geo/query/acc.cgi?acc=GSE76211, we chose |log2FC| > 1 and *P* value <.05 as the cut‐off.

### 2.4 Establishment of outcome signature with LASSO regression model

LASSO (least absolute shrinkage and selection operator) is a regression analysis method that performs both variable selection and regularization in order to enhance the prediction accuracy and interpretability of the statistical model. Here, based on ‘glmnet’ R package, LASSO Cox regression analysis was applied to build an optimal prognostic signature for BCa by using candidate biomarkers.^14^ Since we identified some candidates involved in tumour progression, dataset E‐MTAB‐4321 with progression free survival data was chosen to set up the outcome model. All the patients were randomly divided into 2 groups (70% as the discovery set and 30% as the test set) and LASSO Cox regression model was constructed by using 70% of the patients in discovery set. The optimal values of the penalty parameter lambda were determined through 10‐times cross‐validations. The simplest (smallest parameter) model of prognostic genes signature within one standard error of the best lambda value was screened out. The risk score of prognostic signature for each sample was calculated by the relative expression of each prognostic gene in the signature and its associated coefficient. The risk score of the outcome signature = $\sum_{i=1}^{n}$ (coef_i_ × Expr_i_), where Expr_i_ is the relative expression of the gene in the signature for patient i, coef_i_ is the LASSO coefficient of the gene i. Since no other dataset including progression free survival data for BCa, we only did the internal validation for PFS in E‐MTAB‐4321. Meanwhile, to further validate the clinical use for our model, prediction for overall survival (OS) and disease free survival (DFS) were tested in other independent datasets (http://www.ncbi.nlm.nih.gov/geo/query/acc.cgi?acc=GSE13507, http://www.ncbi.nlm.nih.gov/geo/query/acc.cgi?acc=GSE32894 and http://www.ncbi.nlm.nih.gov/geo/query/acc.cgi?acc=GSE5287). Besides, to prove the wide utility of our model, pan‐cancer outcome validation was performed via KMPlotter database (https://kmplot.com/analysis/).

### 2.5 Estimation of outcome signature for patients' prognosis

Patients from different datasets were equally divided into low‐risk group and high‐risk group due to the risk score of our outcome signature. Then time ‐ dependent receiver operating characteristic curve (ROC) analysis by using ‘survivalROC’ package in R, was used to calculate the area under curve (AUC) for 1‐year, 3‐year and 5‐year PFS, OS and DFS, and check the prediction accuracy for our model.^15^

### 2.6 Construction and assessment of the nomogram

First, univariate and multivariate cox regression analysis was performed to identify the proper terms to build the nomogram. The forest was used to show the *P* value, HR and 95% CI of each variable through ‘forestplot’ R package. We use ‘rms’ and ‘rmda’ packages of R software (version3.5.0) to perform the nomogram, calibration plots and decision curve. Nomogram was applied as a predict device to evaluate the prognosis of patients, which has the ability to generate an individual probability of a clinical event by integrating various prognostic factors. Calibration was performed to evaluate the performance of the 5‐year PFS nomogram. The *x*‐axis indicated nomogram‐predicted survival and the *y*‐axis represented observed outcome, and the 45° line represented the best prediction. Additionally, decision curve analysis (DCA) was also applied to evaluate the clinical utility of the nomogram, which could evaluate and compare prediction models which incorporated clinical consequences. The percentage of threshold probability and the net benefit were plotted on the *x*‐axis and *y*‐axis, respectively.

### 2.7 Gene set enrichment analysis (GSEA) and gene set variation analysis (GSVA)

According to the median risk score in discovery set, we divided the samples into high‐risk and low‐risk groups. GSEA (http://software.broadinstitute.org/gsea/index.jsp) was used to explore biological function of prognostic signature for 2 groups.^16^ Annotated gene sets h.all.v7.0.symbols.gmt were chosen as the reference gene sets. FDR < 0.05 was set as the cut‐off criteria. Meanwhile, GSVA analysis was also performed via ‘GSVA’ package in R to identify the mostly changed pathways in high‐ and low‐risk groups.^17^ *T* value >2 and FDR < 0.05 were chosen as the cut‐off. Then, we merged the common pathways from 2 analyses.

### 2.8 Ethical statement for human bladder tissue samples

Bladder cancer tissue samples and paracancerous tissues (n = 12) were obtained from bladder cancer patients and stored in liquid nitrogen or fixed in 4% paraformaldehyde (PFA) for further analysis. Informed consent was obtained from all subjects. The Ethics Committee at Zhongnan Hospital of Wuhan University approved the experiments using human bladder tissue samples for RNA isolation and immunofluorescence staining analysis (approval number: 2015029). All methods used for human bladder tissue samples were performed in accordance with the approved guidelines and regulations.

### 2.9 RNA isolation, reverse transcription and quantitative real time PCR (qRT‐PCR)

Total RNA from bladder cancer and paracancerous tissues was isolated using RNeasy Mini Kit (Cat. #74101, Qiagen) according to the manufacturer's instruction. DNase I digestion (Cat. #79254, Qiagen) was used in each RNA preparation to remove genomic DNA. After that, the quantity of isolated RNA was measured by a NanoDrop^®^ ND‐1000 UV‐Vis spectrophotometer (Thermo Scientific). The cDNA was synthesized using 1 μg of total RNA isolated from bladder cancer and paracancerous tissues by ReverTra Ace qPCR RT Kit (Toyobo) and qRT‐PCR was performed using 500 ng cDNA per 20 μL reaction. Each reaction was conducted with iQTM SYBR^®^ Green Supermix (Bio‐Rad) using 500 ng of cDNA in a final volume of 20 μL. Primer sequences and annealing temperatures are summarized in Table S6. Values were normalized for amplified GAPDH alleles.

### 2.10 Immunofluorescence staining for human bladder tissue samples

The samples were fixed by 4% PFA at 4°C overnight and embedded into paraffin (Paraplast, Sigma‐Aldrich) by tissue processor (Cat. #STP 120, Thermo Fisher Scientific). Then the rotation microtome (Cat. #HM325, Thermo Fisher Scientific) was used to cut paraffin sections (4 µm). The sections were incubated with indicated primary antibody and FITC‐labelled secondary antibody in humidified atmosphere (antibodies listed in Table S5). Nuclei were labelled with DAPI (2 μg/mL). The results were photographed by fluorescence microscope.

### 2.11 Statistical analysis

R software 3.5.0 was used for all statistical analyses. Statistical significance was set at probability values of *P* < .05. Two‐tailed Student's *t* test was used for significance of differences between subgroups. One‐way ANOVA test or Student *t* test were applied to analyse the correlation between risk score and clinicopathological parameters. Kaplan‐Meier survival curves were built to analyse survival differences between the high‐risk group and low‐risk group. The ROC, calibration curve and DCA were compared for the predictive accuracy of the prognostic models.

## 3 RESULTS

### 3.1 Selection of candidate genes via WGCNA analysis and differentially expressed genes screening

**Figure 2:**
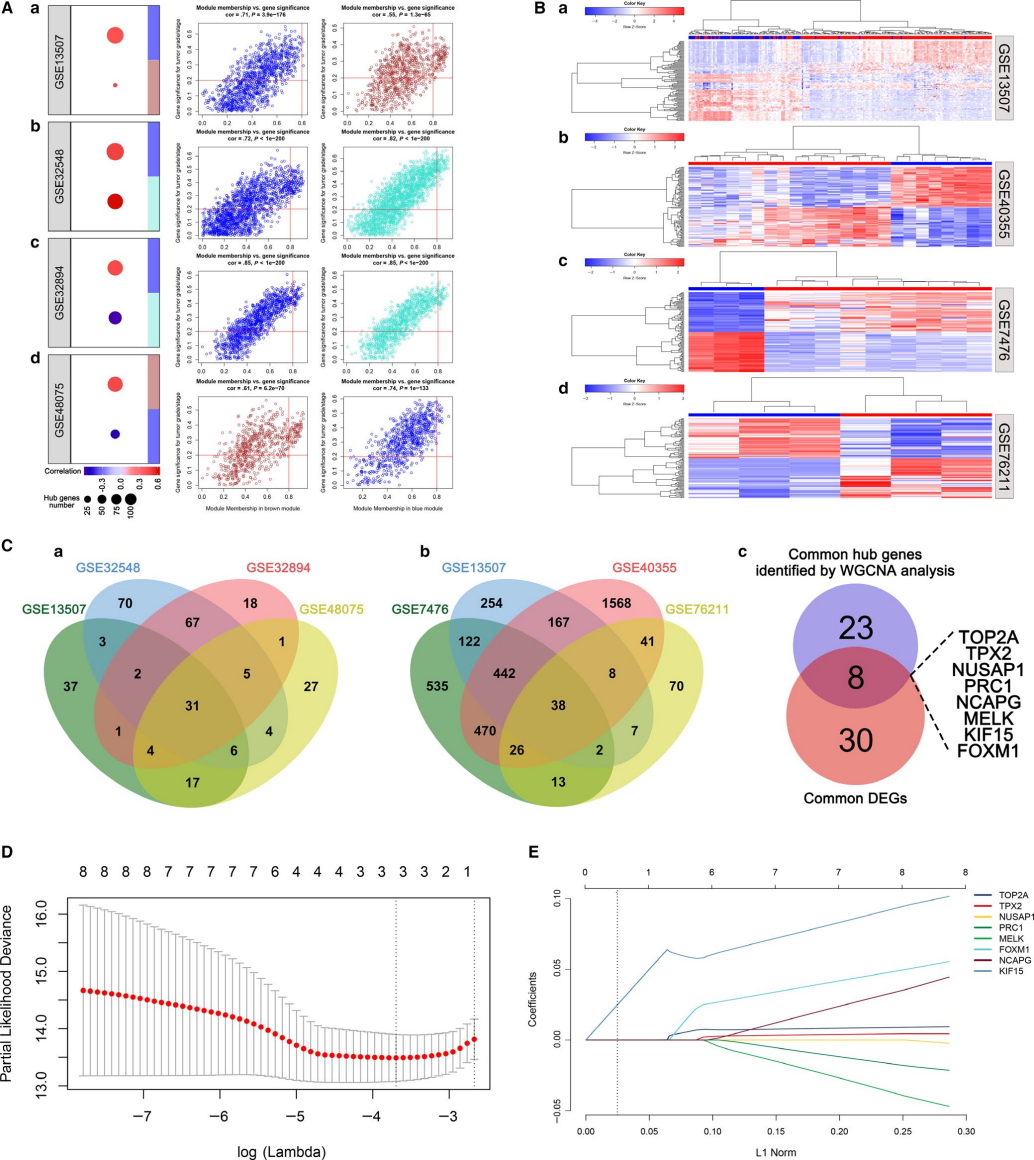
The construction of LASSO Cox regression model. A, Scatter plot of progression‐related models identified by WGCNA in http://www.ncbi.nlm.nih.gov/geo/query/acc.cgi?acc=GSE13507 (a), http://www.ncbi.nlm.nih.gov/geo/query/acc.cgi?acc=GSE32548 (b), http://www.ncbi.nlm.nih.gov/geo/query/acc.cgi?acc=GSE32894 (c) and http://www.ncbi.nlm.nih.gov/geo/query/acc.cgi?acc=GSE48075 (d). B, DEGs analysis of http://www.ncbi.nlm.nih.gov/geo/query/acc.cgi?acc=GSE7476 (a), http://www.ncbi.nlm.nih.gov/geo/query/acc.cgi?acc=GSE13507 (b), http://www.ncbi.nlm.nih.gov/geo/query/acc.cgi?acc=GSE40355 (c) and http://www.ncbi.nlm.nih.gov/geo/query/acc.cgi?acc=GSE76211 (d). C, Venn diagram shows the intersection genes of http://www.ncbi.nlm.nih.gov/geo/query/acc.cgi?acc=GSE13507, http://www.ncbi.nlm.nih.gov/geo/query/acc.cgi?acc=GSE32548, http://www.ncbi.nlm.nih.gov/geo/query/acc.cgi?acc=GSE32894 and http://www.ncbi.nlm.nih.gov/geo/query/acc.cgi?acc=GSE48075 (a) and http://www.ncbi.nlm.nih.gov/geo/query/acc.cgi?acc=GSE7476, http://www.ncbi.nlm.nih.gov/geo/query/acc.cgi?acc=GSE13507, http://www.ncbi.nlm.nih.gov/geo/query/acc.cgi?acc=GSE40355 and http://www.ncbi.nlm.nih.gov/geo/query/acc.cgi?acc=GSE76211 (b). The intersection genes of hub genes and DEGs (c). D and E, Construction of LASSO Cox regression model in E‐MTAB‐4321

Based on WGCNA analysis, we identified 2 key modules for each dataset (Figures S1 and S2 and Table S2) and eventually obtained 101, 188, 129 and 95 hub genes from http://www.ncbi.nlm.nih.gov/geo/query/acc.cgi?acc=GSE13507, http://www.ncbi.nlm.nih.gov/geo/query/acc.cgi?acc=GSE32548, http://www.ncbi.nlm.nih.gov/geo/query/acc.cgi?acc=GSE32894 and http://www.ncbi.nlm.nih.gov/geo/query/acc.cgi?acc=GSE48075 respectively (Figure 2A). Meanwhile, we found 1040, 1650, 2760 and 205 genes were differentially expressed between tumour and non‐tumour samples in http://www.ncbi.nlm.nih.gov/geo/query/acc.cgi?acc=GSE13507, http://www.ncbi.nlm.nih.gov/geo/query/acc.cgi?acc=GSE7476, http://www.ncbi.nlm.nih.gov/geo/query/acc.cgi?acc=GSE40355 and http://www.ncbi.nlm.nih.gov/geo/query/acc.cgi?acc=GSE76211 (Figure 2B and Table S3). Then we intersected them separately to produce 31 hub genes and 38 DEGs (Figure 2C). Finally, 8 candidate genes (TOP2A, TPX2, NUSAP1, PRC1, MELK, KIF15 and FOXM1) were generated by taking the intersection of them (Figure 2C).

### 3.2 Construction of outcome model

70% of the patients in dataset E‐MTAB‐4321 were used as the discovery set and 8 candidate genes were used to construct the LASSO Cox regression model. We applied a 10‐fold cross validation to select an optimal value and identify three genes: TOP2A, TPX2 and NCAPG (Figure 2D,E). Then, a formula was generated according to the expression of the three genes to predict the risk score of each individual BCa patient, where risk score = 1.896E−03 * Exp_TOP2A_ + 4.784E−02 * Exp_TPX2_ + 2.850E−03 * Exp_NCAPG_.

### 3.3 Validation of prognostic model

**Figure 3:**
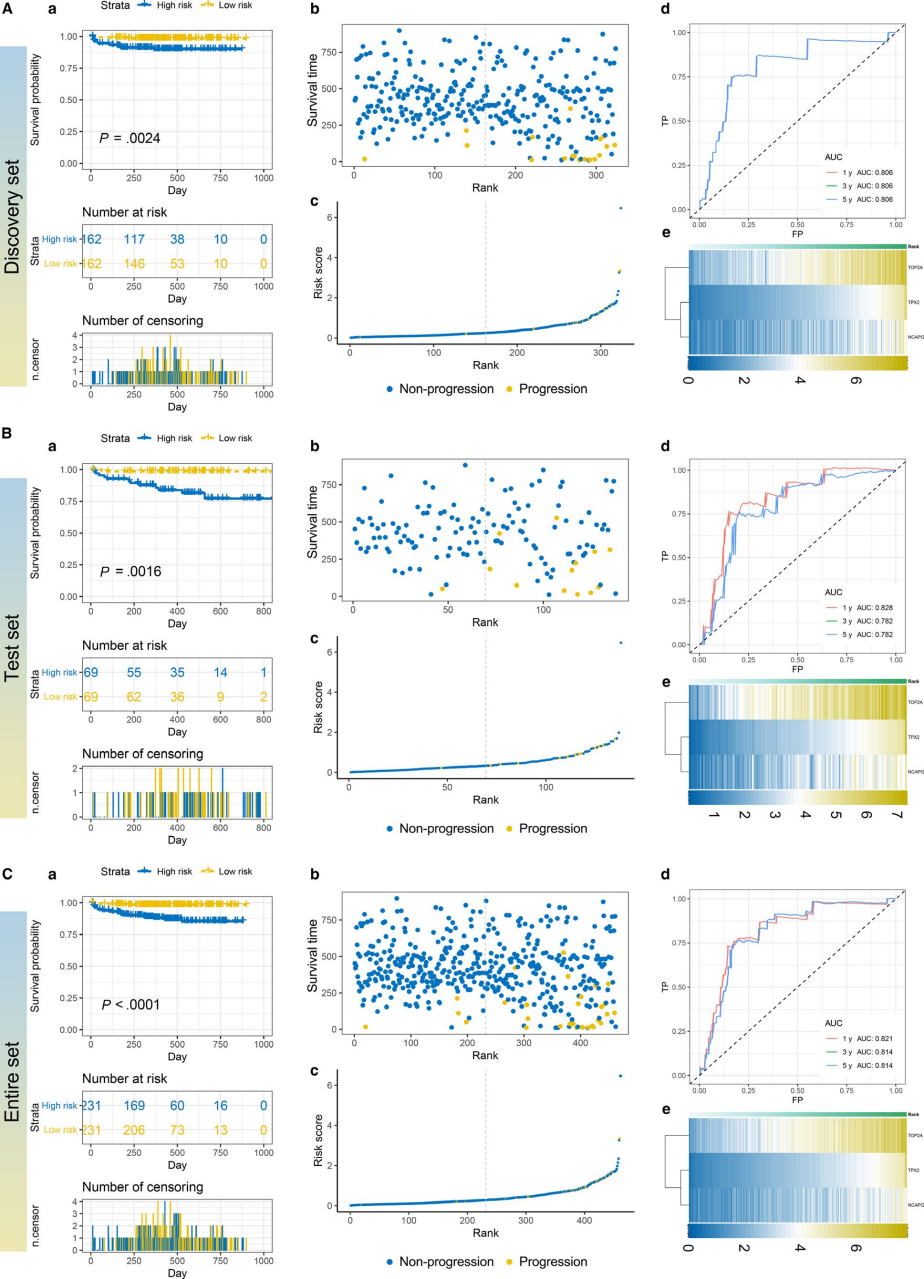
The performance of prognostic model in predicting PFS. A‐C, Kaplan–Meier survival analysis (a), relationship between survival status ‐ risk score rank and survival time (day) ‐ risk score rank (b, c), time ‐ dependent ROC curve for PFS, the AUC was assessed at 1, 3 and 5 y (d), and heat map of three candidates (e)

Patients were divided into high‐risk and low‐risk group based on median risk score. In discovery set, the PFS of patients in higher risk group were obviously worse than the low‐risk group (Figure 3A). Time‐dependent ROC analysis showed that the prediction accuracy of the outcome model at 1, 3, and 5 years remained the same and AUC was 0.806. (Figure 3A). Additionally, the performance of this prognostic model in the prediction of PFS were validated in the test set and entire set of E‐MTAB‐4321 and the results were consistent with the discovery set; with the increasing number of the patients, our outcome model showed higher significance and higher AUC (Figure 3B,C). Besides, with the increasing risk score, expression of 3 candidate genes in our outcome model also increased (Figure 3A‐C).

**Figure 4:**
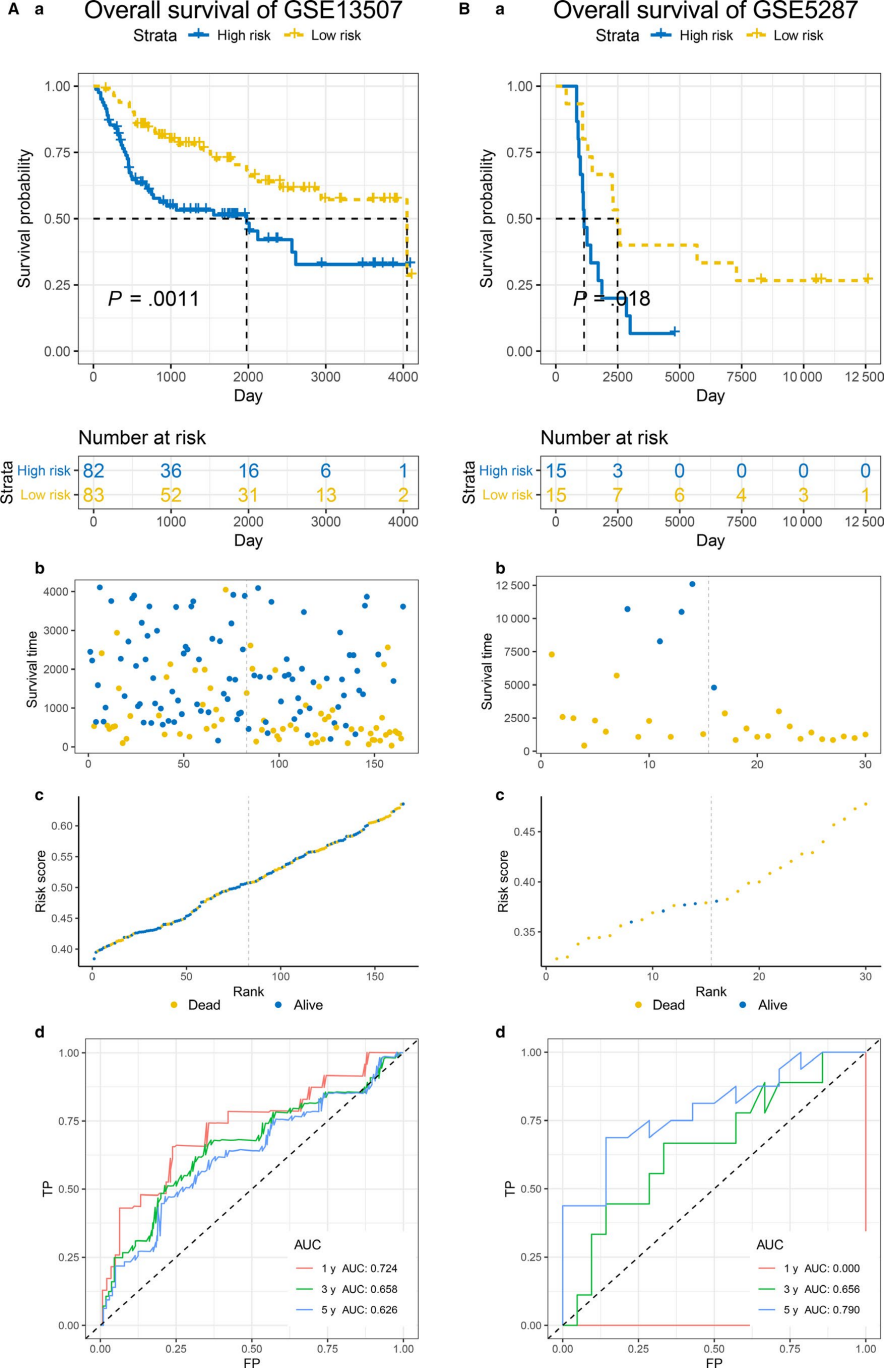
The validation of prognostic model in predicting OS. A and B, Kaplan–Meier survival analysis (a), relationship between survival status ‐ risk score rank and survival time (day) ‐ risk score rank (b, c) and time‐dependent ROC curves for OS (d) in http://www.ncbi.nlm.nih.gov/geo/query/acc.cgi?acc=GSE13507 and http://www.ncbi.nlm.nih.gov/geo/query/acc.cgi?acc=GSE5287

**Figure 5:**
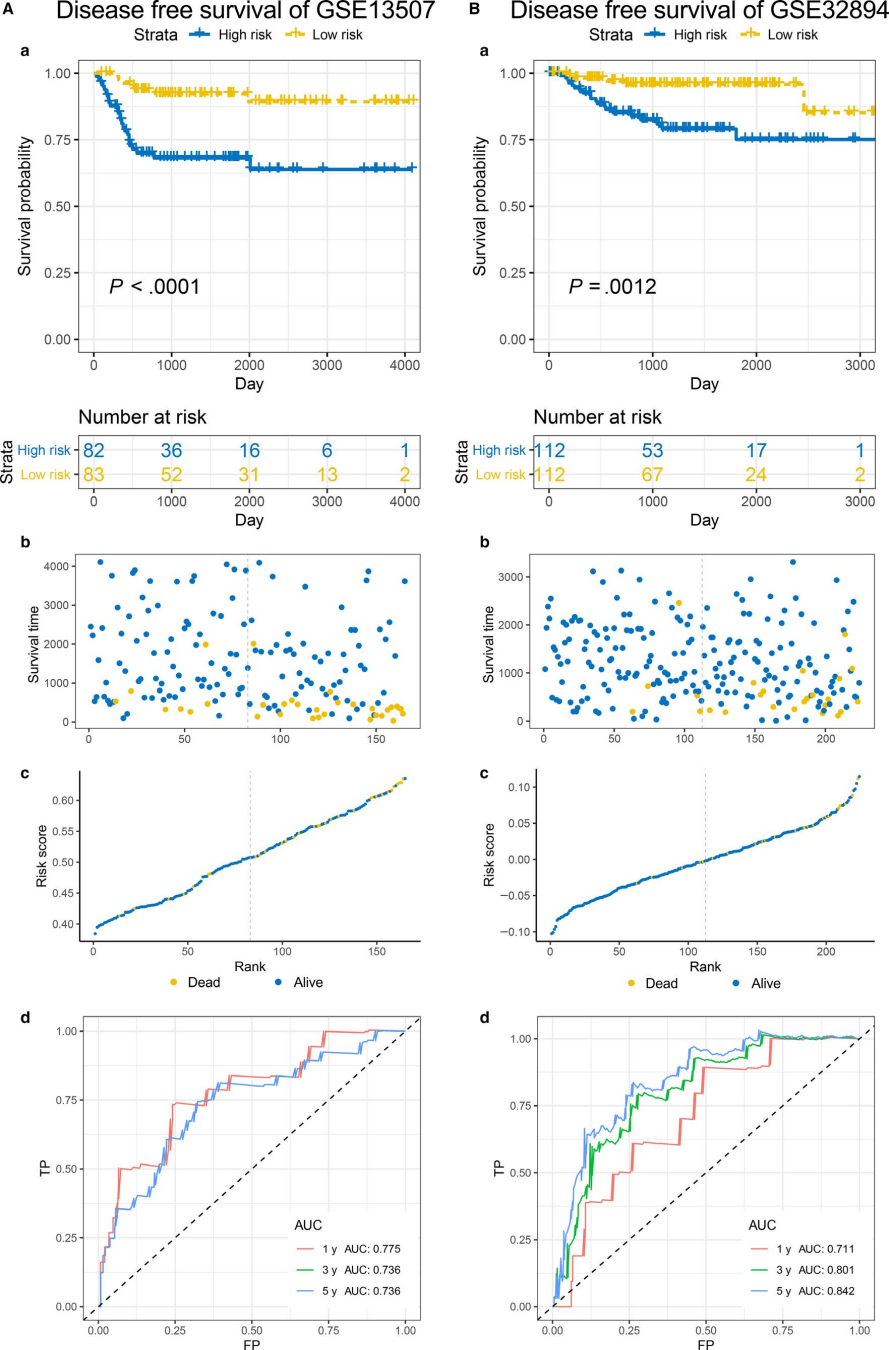
The validation of prognostic model in predicting DFS. A and B, Kaplan–Meier survival analysis (a), relationship between survival status ‐ risk score rank and survival time (day) ‐ risk score rank (b, c) and time‐dependent ROC curves for DFS (d) in http://www.ncbi.nlm.nih.gov/geo/query/acc.cgi?acc=GSE13507 and http://www.ncbi.nlm.nih.gov/geo/query/acc.cgi?acc=GSE32894

Due to lack of external validation for PFS, instead, here we utilized datasets http://www.ncbi.nlm.nih.gov/geo/query/acc.cgi?acc=GSE13507 and http://www.ncbi.nlm.nih.gov/geo/query/acc.cgi?acc=GSE5287 to validate the effectiveness for the prediction of OS. In these two cohorts, the patients in the high‐risk group also show worse OS than the patients in low‐risk group (Figure 4A,B) (*P* value: .0011 in http://www.ncbi.nlm.nih.gov/geo/query/acc.cgi?acc=GSE13507 and .018 in http://www.ncbi.nlm.nih.gov/geo/query/acc.cgi?acc=GSE5287). As for the prediction accuracy for the OS, the 1‐, 3‐, and 5‐year AUC were 0.724, 0.658 and 0.626 in http://www.ncbi.nlm.nih.gov/geo/query/acc.cgi?acc=GSE13507 and none, 0.656 and 0.790 in http://www.ncbi.nlm.nih.gov/geo/query/acc.cgi?acc=GSE5287, which represented high accuracy for OS prediction (Figure 4A,B). For the DFS, the trend of high‐risk group was still worse than the low‐risk group in http://www.ncbi.nlm.nih.gov/geo/query/acc.cgi?acc=GSE13507 (*P* value <.0001) and http://www.ncbi.nlm.nih.gov/geo/query/acc.cgi?acc=GSE32894 (*P* value = .0012) (Figure 5A,B) and the AUC was 0.775, 0.711 at 1‐year; 0.736, 0.801 at 3‐year; 0.736, 0.842 at 5‐year, respectively (Figure 5A,B), which demonstrated a dramatic applicability of our outcome model for DFS prediction.

### 3.4 The performance of prognostic model in pan‐cancer

The performance of the prognostic model has been validated in different datasets of BCa. Besides, we validated this model in various cancers and that the high‐risk group of most tumours showed a worse prognosis including renal clear cell carcinoma, renal papillary cell carcinoma, lung adenocarcinoma, hepatocellular carcinoma, pancreatic ductal adenocarcinoma, pheochromocytoma and paraganglioma, sarcoma, thyroid carcinoma and uterine corpus endometrial carcinoma (Figure S4A‐I). In contrast, the high‐risk group of thymoma and stomach adenocarcinoma showed a better prognosis than low‐risk group (Figure S4J,K).

### 3.5 Correlation between the outcome model with other clinicopathological characters

Clinicopathological data, including age, gender, histological subtype, grade, tumour size, tumour stage and BCG treatment, were collected from E‐MTAB‐4321 dataset. The detailed information of patients' clinicopathological characters in our E‐MTAB‐4321 cohort were displayed in the Table S4. Comparison results between risk score and different clinicopathological characters were shown in Figure S3.

### 3.6 Subgroup analysis of prognostic value of the outcome model

**Figure 6:**
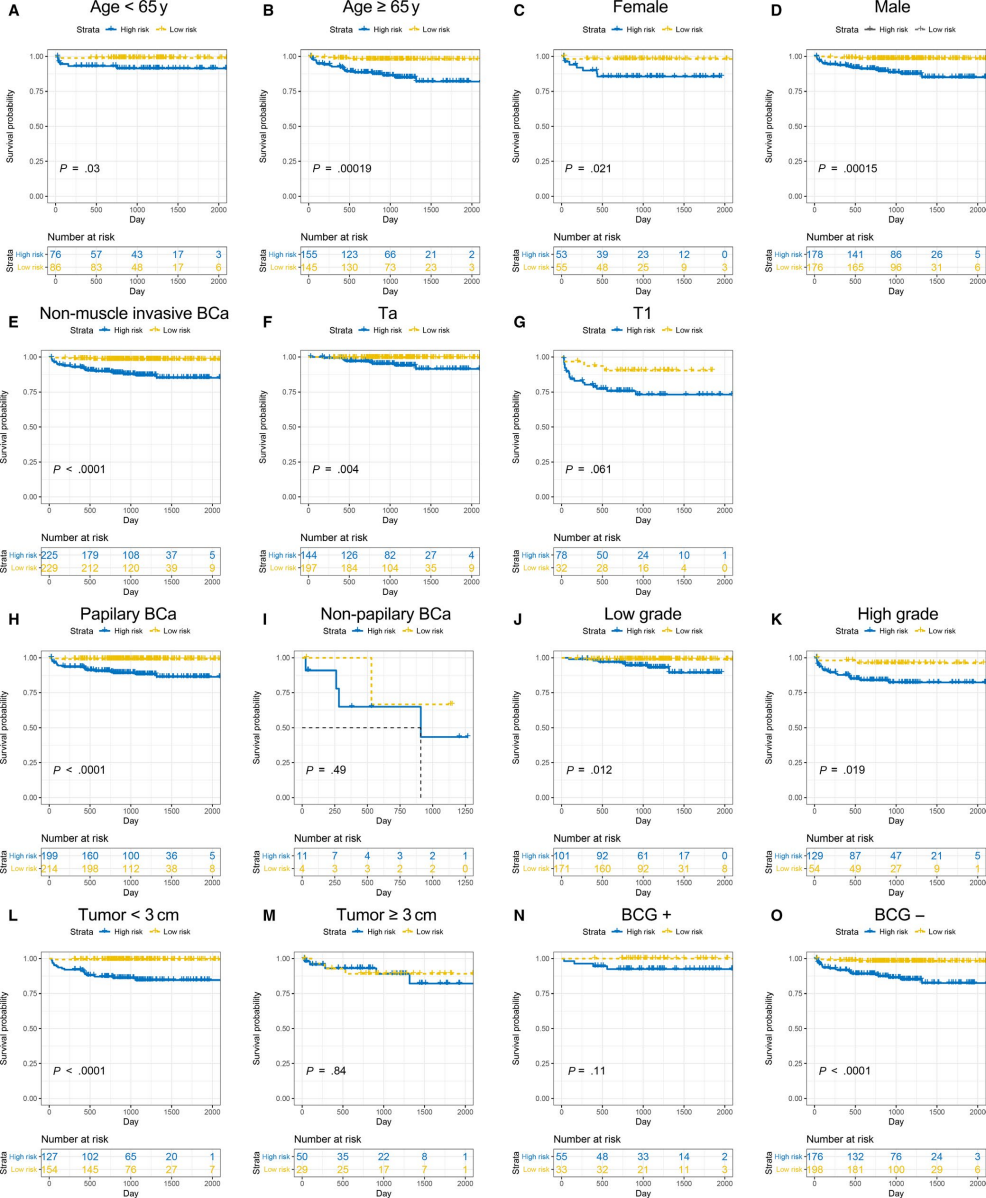
Subgroup analysis of prognostic model in E‐MTAB‐4321. The Kaplan‐Meier survival analysis for age < 65 y (A), age > 65 y (B), female (C) male (D), non‐muscle invasion BCa (E), Ta (F) and T1 (G), papillary (H), non‐papillary (I), low grade (J), high grade (K), tumour < 3 cm (L), and tumour > 3 cm (M), BCG irrigation (N) and non‐BCG irrigation (O)

Prognostic models still show good applicability in subgroup analysis. The prognosis of the high‐risk group in different age and gender subgroups was still worse than the low‐risk group (Figure 6A‐D). Simultaneously, we divided patients into different subgroup according to tumour type, size, and stage. Except for T1, non‐papillary BCa and tumour >3 cm subgroup, all subgroups indicates a poor PFS in the high‐risk group (Figure 6E‐M). Interestingly, in the non‐BCG irrigation subgroup, we could observe that more high‐risk patients suffered BCG treatment, although the high‐risk group had a poorer prognosis trend and there was no significant difference (Figure 6N,O).

### 3.7 Univariate and multivariate cox regression analyses for the model prognostic ability

We performed univariate and multivariate cox regression analysis to investigate whether our model was a clinically independent prognostic factor for BCa patients. And from the unicox regression analysis, tumour grade, tumour stage, age, tumour subtype and risk score were significant, as too many missing patients were in subtype group, although it showed a significant trend, we still removed it from the multicox regression analysis (Figure S5). Eventually, the results revealed that in our outcome model, tumour stage and risk score were the only two independent prognostic factors to predict progression free survival rate in BCa patients; however, to some extent, age could be somehow a valuable parameter (Figure S5).

### 3.8 Construction of nomogram

**Figure 7:**
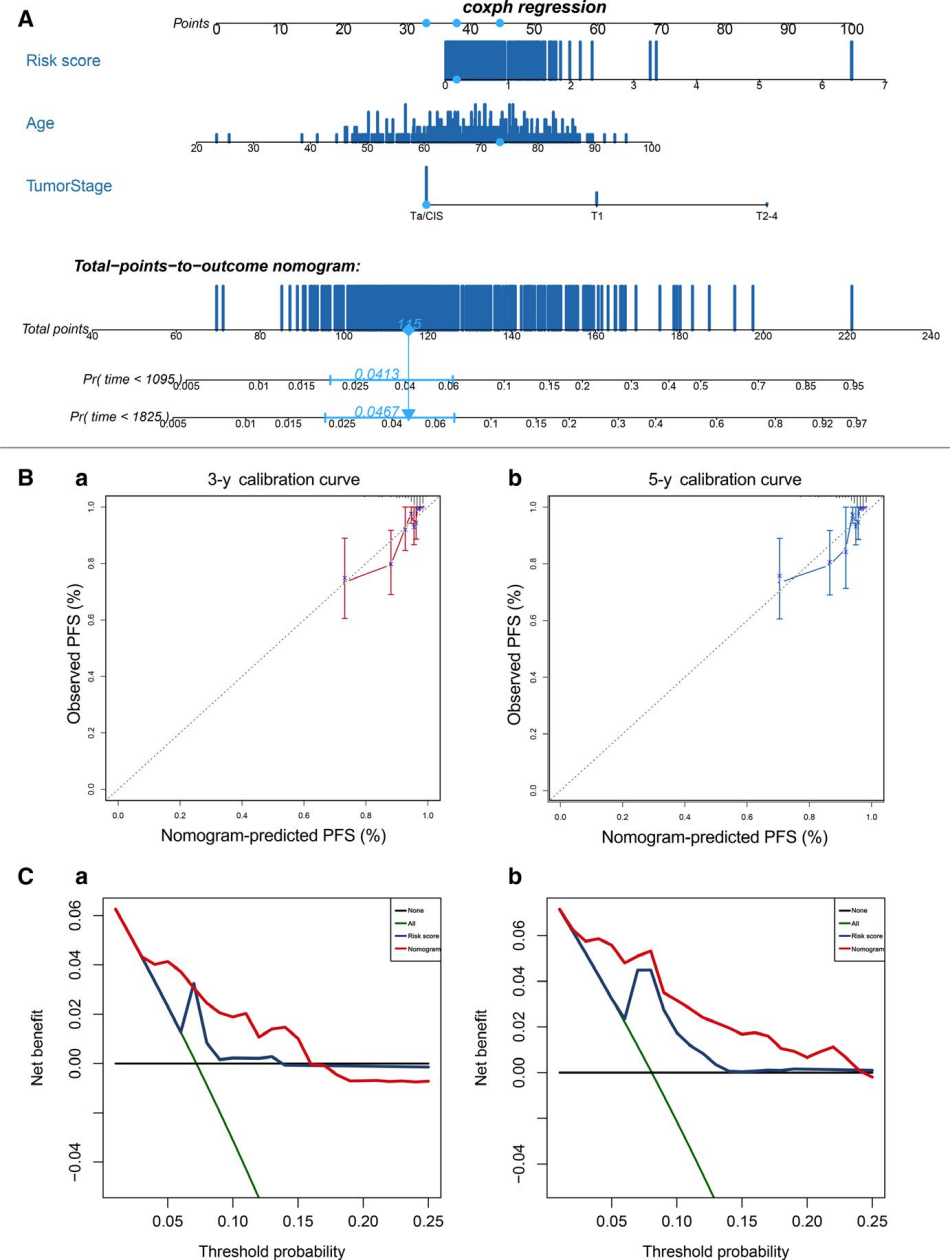
The construction of nomogram. A, The construction of nomogram. B, The calibration plots for predicting patient 3‐year (a) or 5‐year (b) PFS. Nomogram‐predicted PFS is plotted on the *x*‐axis; observed PFS is plotted on the *y*‐axis. C, DCA for assessment of the clinical utility of the nomogram. The *x*‐axis indicates the percentage of threshold probability, and the *y*‐axis indicates the net benefit

According to the results of univariate and multivariate cox regression analysis, we further construct a nomogram combining the only two independent prognostic factors, including the risk score and tumour stage, and one potential factor patient's age to provide a quantitative method for the clinicians to predict the probability of 3‐year and 5‐year PFS in BCa patients (Figure 7A). Every patient would get a total point by plus each prognostic parameters point, and the higher total points mean a worse outcome for that patient. Moreover, the calibration curve indicated that good performance in the estimation of 3‐year and 5‐year PFS of the nomogram compared with the estimation of Kaplan‐Meier (Figure 7B). The results of DCA analysis also demonstrated that our nomogram was of high potential for clinical usefulness (Figure 7C).

### 3.9 Functional enrichment analysis

**Figure 8:**
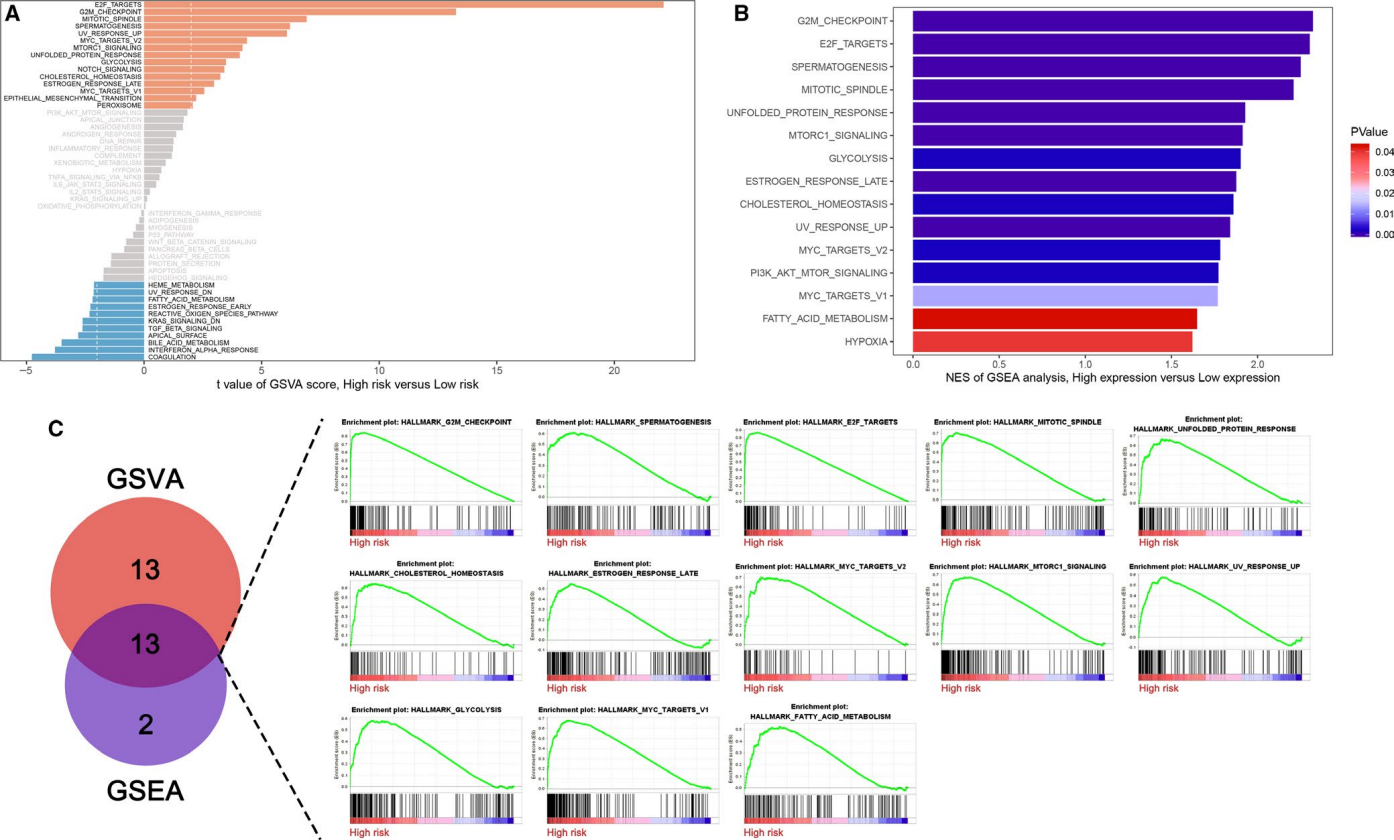
Three gene signatures related pathway enrichment analysis. A, The GSVA analysis shows the most related 26 pathways. B, GSEA analysis shows the most related 15 pathways. C, The 13 intersection pathways between GSVA and GSEA

The GSVA analysis showed that the three gene signatures were highly correlated with 26 pathways and another 15 pathways was enriched by GSEA for the three gene signatures (Figure 8A,B and Table S7 and Table S8). After intersecting the two pathway groups, the most related 13 pathways were showed in Figure 8C.

### 3.10 Gene expression validation

First, we used the database to prove the significantly dysregulated expression of three candidates between tumour and non‐tumour and different stages BCa (Figure S6A,B). Then immunofluorescence and qRT‐PCR of BCa and paracancerous tissues were performed, which also showed a significant up‐regulation role of those three in cancer tissues (Figure S6C,D).

## 4 DISCUSSION

Last thirty years, there is no significant improvement in the diagnostics, treatment and five‐year survival rates of BCa.^18^ To monitoring the recurrence and progression of BCa, the patients had to undergo cystoscopy after surgery, which will bring a huge mental and economic burden to the patient.^19^, ^20^ Our group has previously found some biomarkers related to BCa prognosis and carcinogenesis.^21^, ^22^, ^23^ These biomarkers may contribute to the prediction of prognosis for BCa patients. Nevertheless, a single biomarker lacks sufficiently accuracy and effectiveness, the comprehensive methods combined several biomarkers can significantly improve the accuracy and effectiveness. Our outcome model was the first prognosis model for human bladder cancer progression prediction via integrative bioinformatics analysis.

In this study, we performed WGCNA and differentially expressed gene screening on seven datasets and the intersection eight candidate genes were obtained. Based on the eight genes, the outcome model including three genes was constructed to predict the progression of human BCa in E‐MTAB‐4321. Simultaneously, the patients were divided into high‐ and low‐risk groups based on the median risk score. Then, discovery set, test set and entire set were used to validate the performance of the outcome model and the results show high‐risk group had a worse PFS than low‐risk group. We also validated the effectiveness of model in predicting OS and DFS in three independent datasets. Furthermore, the performance of the model went through pan‐cancer validation by KMPlotter database. In addition, the nomogram based on the model exhibits an impressive performance and clinical applicability.

The genes (TOP2A, TPX2, NCAPG) in our prognostic model have been previously reported to be associated with BCa. Topoisomerase‐II alpha (TOP2A) is essential enzyme for chromosome condensation and chromatid separation by regulating DNA topological state in the process of replication and transcription.^24^ Several previously publications have uncovered that TOP2A is a poor prognostic biomarker in BCa.^25^, ^26^, ^27^ In addition, Zeng et al^28^ demonstrated that TOP2A is positive correlated with the metastasis and tumorigenesis of BCa. The targeting protein for Xenopus kinesin‐like protein 2 (TPX2) is a spindle assembly related protein which play an essential role in cell division. It has been reported that overexpression TPX2 can suppress cell proliferation and invasion and can predict a poor survival in BCa.^29^, ^30^ Non–structural maintenance of chromosomes condensin I complex subunit G (NCAPG) can active the condensin complex to promote the meiosis and mitosis.^31^ Most of previous publications proved that NCAPG can affect cell proliferation and invasion in prostate cancer and hepatocellular carcinoma. Interestingly, Pan et al proved the important function of NCAPG in maintain BCa stem cell characteristics.

In conclusion, our study revealed that the prognostic model based on comprehensive study including WGCNA, DEGs screening and LASSO Cox regression has reliable reproducibility and accuracy in predicting prognosis of BCa and even pan‐cancer.

## CONFLICT OF INTEREST

The authors declare that there is no conflict of interests.

## AUTHOR CONTRIBUTIONS

GW, LJ, YX and XW conceived and designed the study. YX, LY and JX provided bioinformatics and statistical analysis. HX and YL collected and organized specimens with clinical parameters. All authors have approved this manuscript.

## Data Availability

The datasets used and/or analysed during the current study are available from the corresponding authors on reasonable request.
