# Supplementary material for: An outcome model for human bladder cancer: A comprehensive study based on weighted gene co‐expression network analysis
Source: J Cell Mol Med. 2019 Dec 28;24(3):2342–55. doi: 10.1111/jcmm.14918 (PMC7011142; doi:10.1111/jcmm.14918)
Supplement: Supplementary file 1 [file JCMM-24-2342-s001.docx]

**
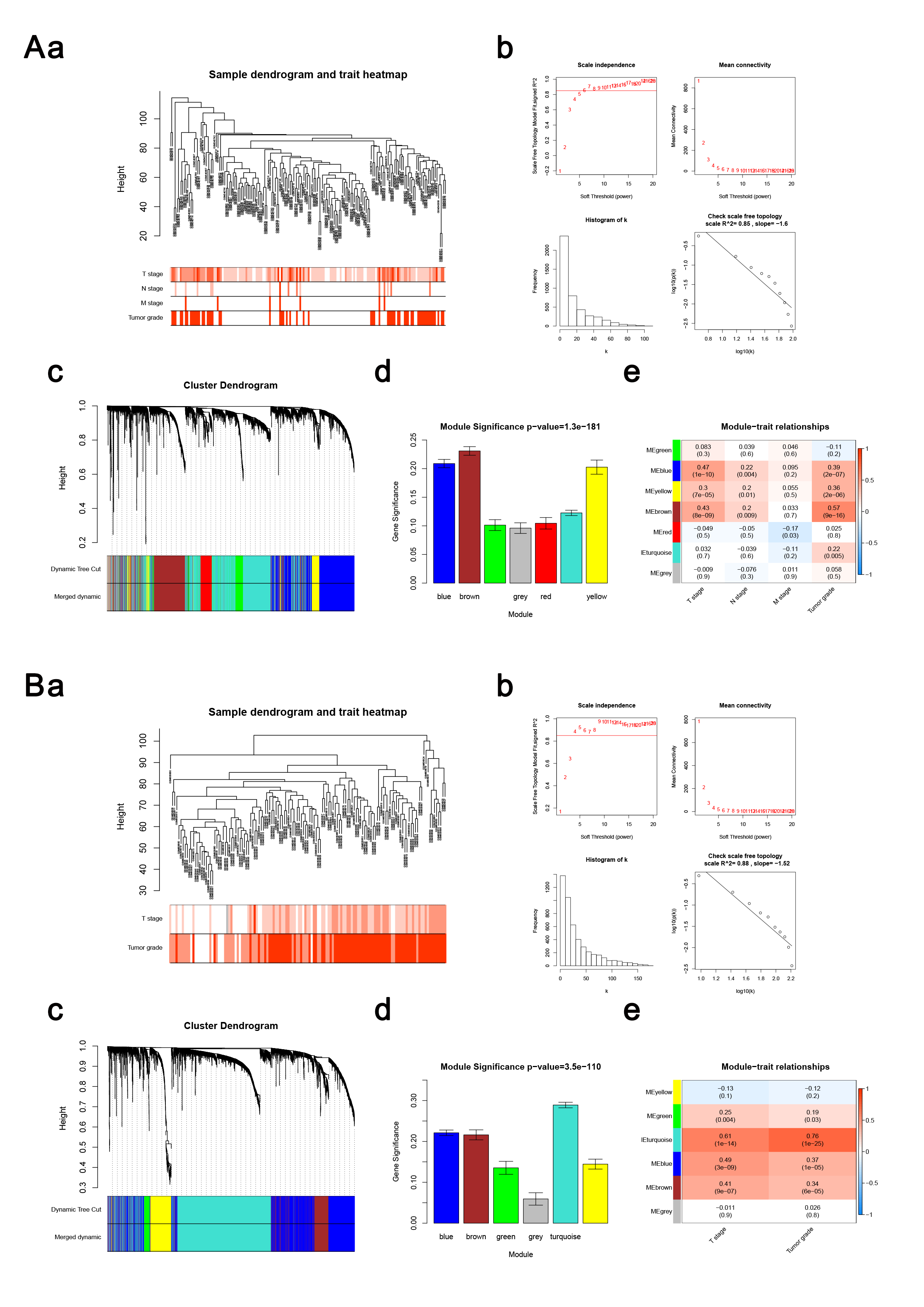
**

**Supplementary Figure S1. WGCNA of GSE13507 and GSE32548. A.** WGCNA of GSE13507. (**a**) Sample dendrogram and Tumor grade heatmap. (**b**) Analysis of the scale-free fit index and the mean connectivity for various soft-thresholding powers (upper). Histogram of connectivity distribution and the scale-free topology (down). (**c**) Dendrogram of all differentially expressed genes clustered. (**d**) Distribution of average gene significance in the modules. (**e**) The correlation between module eigengenes tumor grade. **B.** WGCNA of GSE32548. (**a**) Sample dendrogram and Tumor grade heatmap. (**b**) Analysis of the scale-free fit index and the mean connectivity for various soft-thresholding powers (upper). Histogram of connectivity distribution and the scale-free topology (down). (**c**) Dendrogram of all differentially expressed genes clustered. (**d**) Distribution of average gene significance in the modules. (**e**) The correlation between module eigengenes and tumor grade.


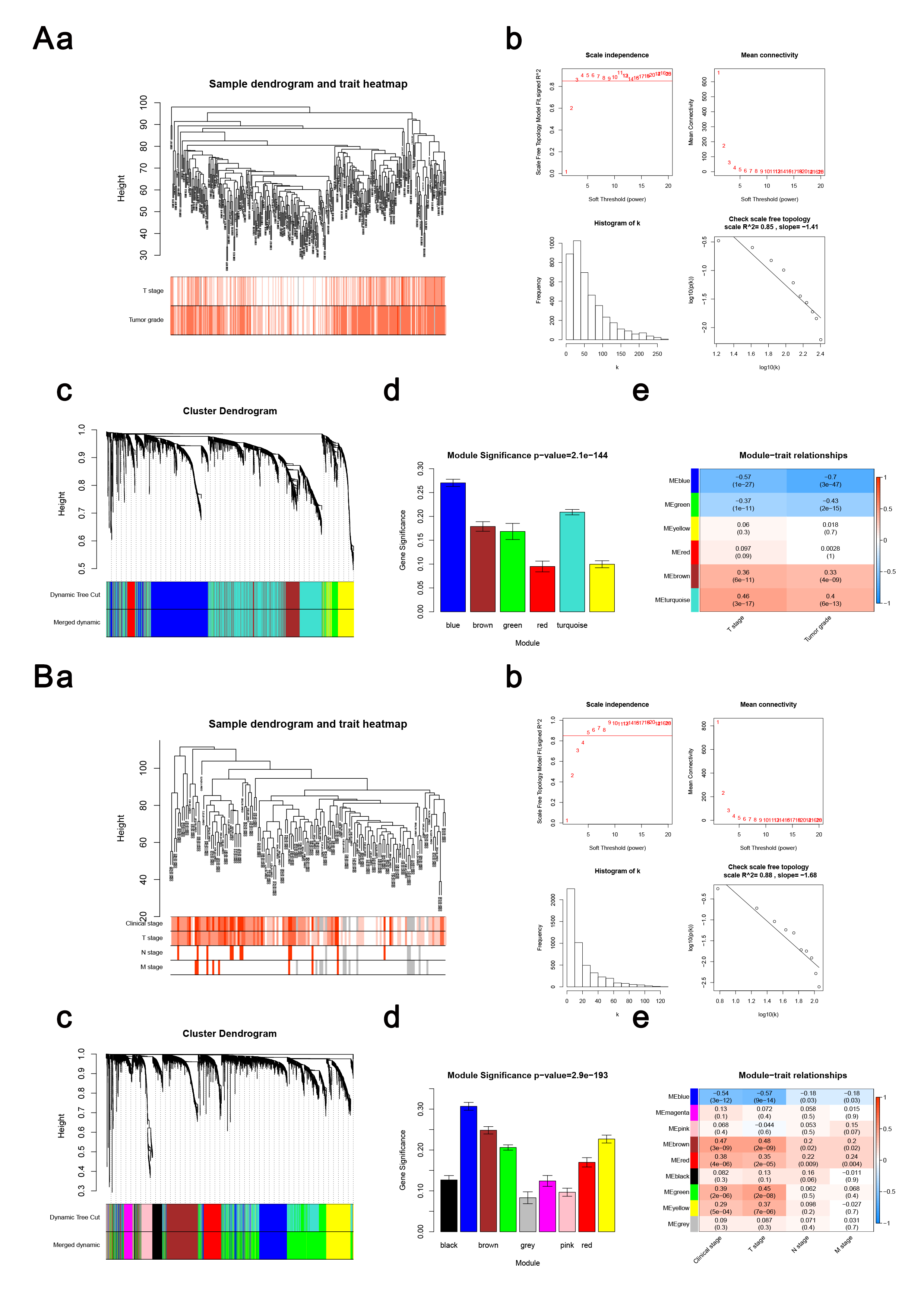


**Supplementary Figure S2. WGCNA of GSE31894 and GSE48075. A.** WGCNA of GSE31894. (**a**) Sample dendrogram and Tumor grade heatmap. (**b**) Analysis of the scale-free fit index and the mean connectivity for various soft-thresholding powers (upper). Histogram of connectivity distribution and the scale-free topology (down). (**c**) Dendrogram of all differentially expressed genes clustered. (**d**) Distribution of average gene significance in the modules. (**e**) The correlation between module eigengenes tumor grade. **B.** WGCNA of GSE48075. (**a**) Sample dendrogram and Tumor grade heatmap. (**b**) Analysis of the scale-free fit index and the mean connectivity for various soft-thresholding powers (upper). Histogram of connectivity distribution and the scale-free topology (down). (**c**) Dendrogram of all differentially expressed genes clustered. (**d**) Distribution of average gene significance in the modules. (**e**) The correlation between module eigengenes and tumor grade.


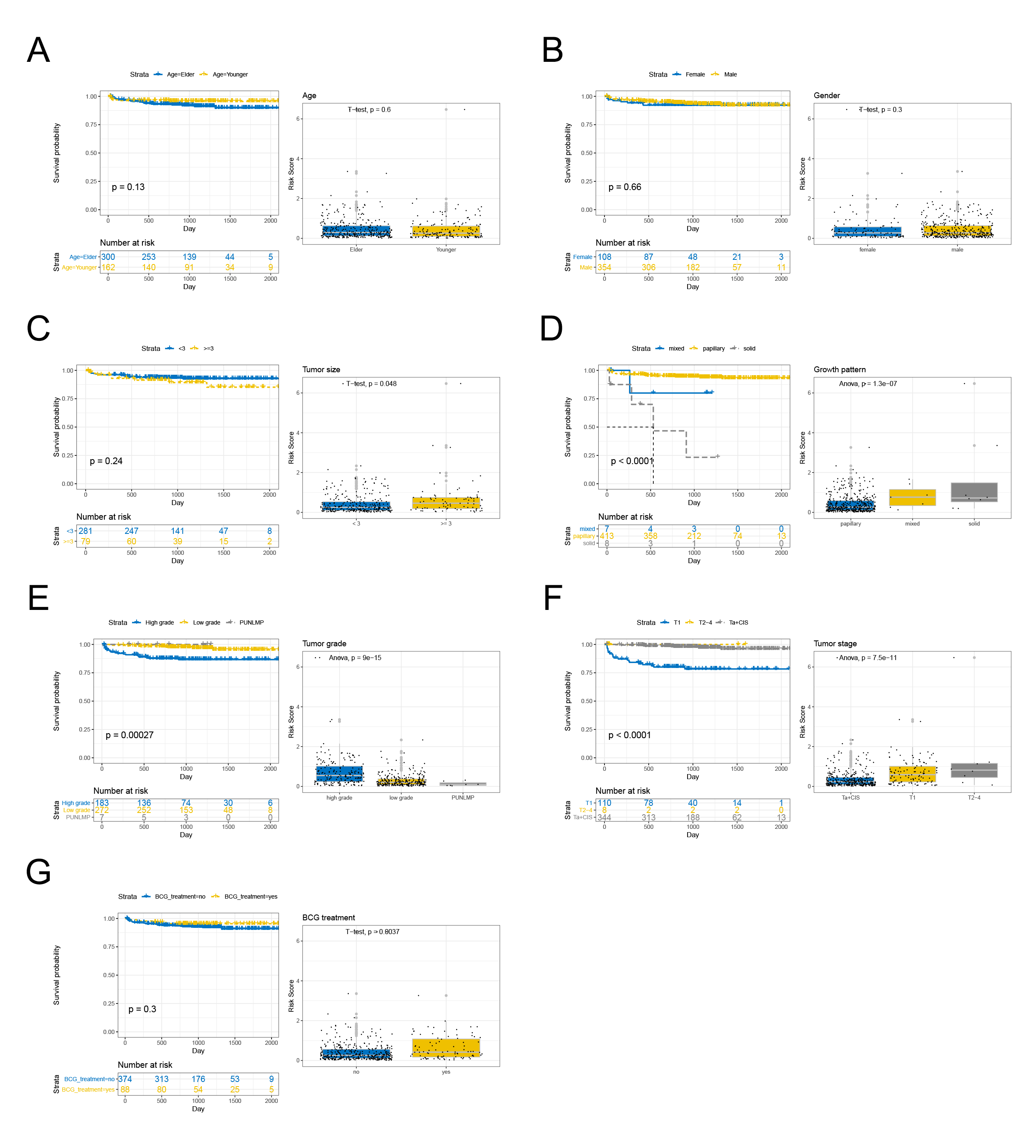


**Supplementary Figure S3. Association between the risk score of outcome model and clinicopathological characters.** The survival rate of indicated subtypes in different clinicopathological characters was measured. Boxplots indicate the correlation between the risk score and the indicated subtype of each clinicopathological character by t - test or one - way Anova test. The patients are stratified into different subgroups based on age: elder: age ≥ 65, younger: age < 65 (**A**); gender: female and male (**B**); tumor size: size ≥ 3cm and size < 3cm (**C**); growth pattern: papillary and non-papillary (solid and mixed) (**D**); tumor grade: PUNLMP, low grade and high grade (**E**); tumor stage: Ta+CIS, T1 and T2-4 (**F**); BCG treatment: yes and no (**G**).


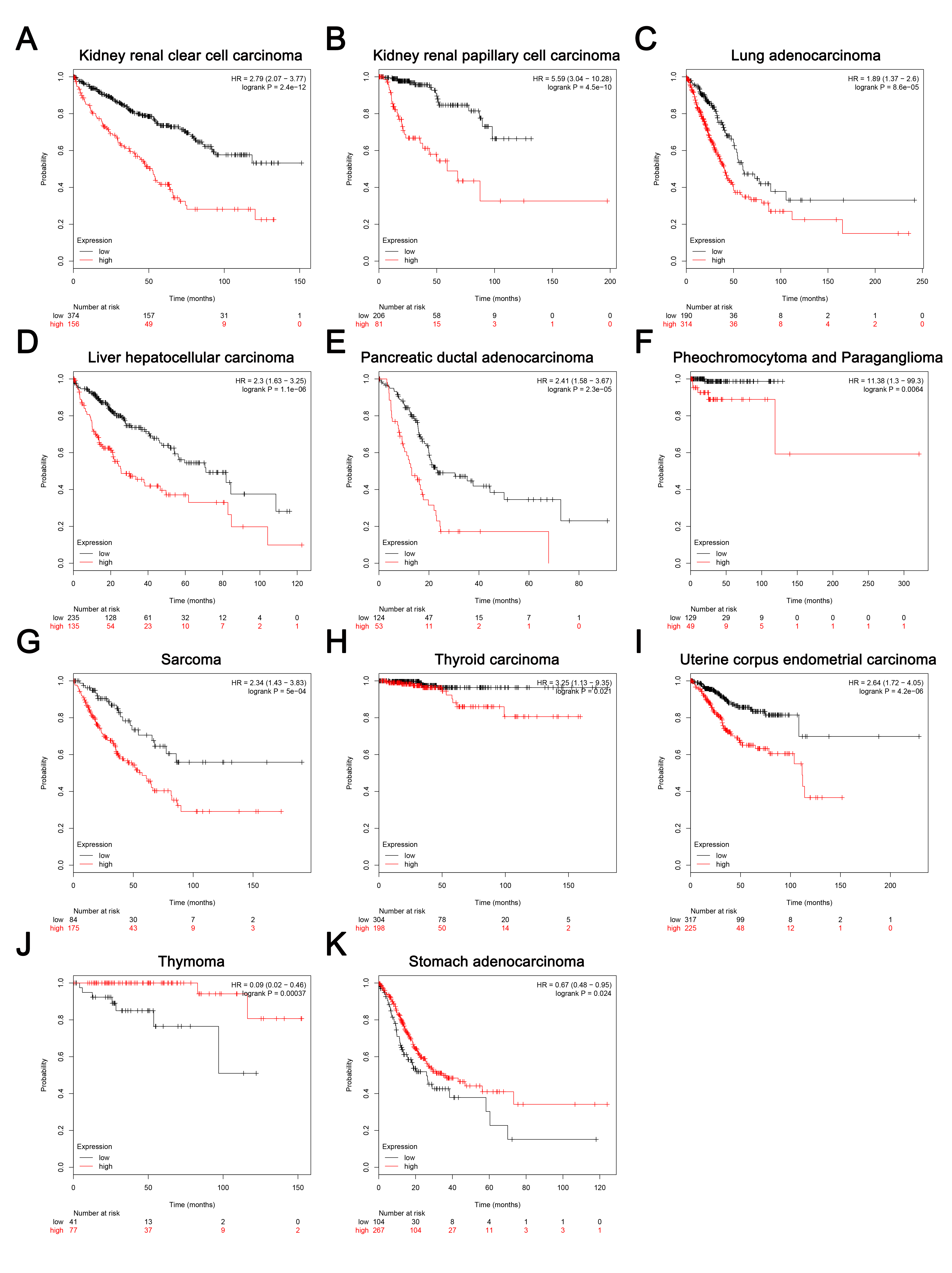


**Supplementary Figure S4. Validation of prognostic model in pan-cancer.** Kaplan-Meier survival analysis in renal clear cell carcinoma (**A**), renal papillary cell carcinoma (**B**), lung adenocarcinoma (**C**), hepatocellular carcinoma (**D**), pancreatic ductal adenocarcinoma, (**E**) pheochromocytoma and paraganglioma (**F**), sarcoma (**G**), thyroid carcinoma (**H**), uterine corpus endometrial carcinoma (**I**), thymoma (**J**) and stomach adenocarcinoma (**K**).


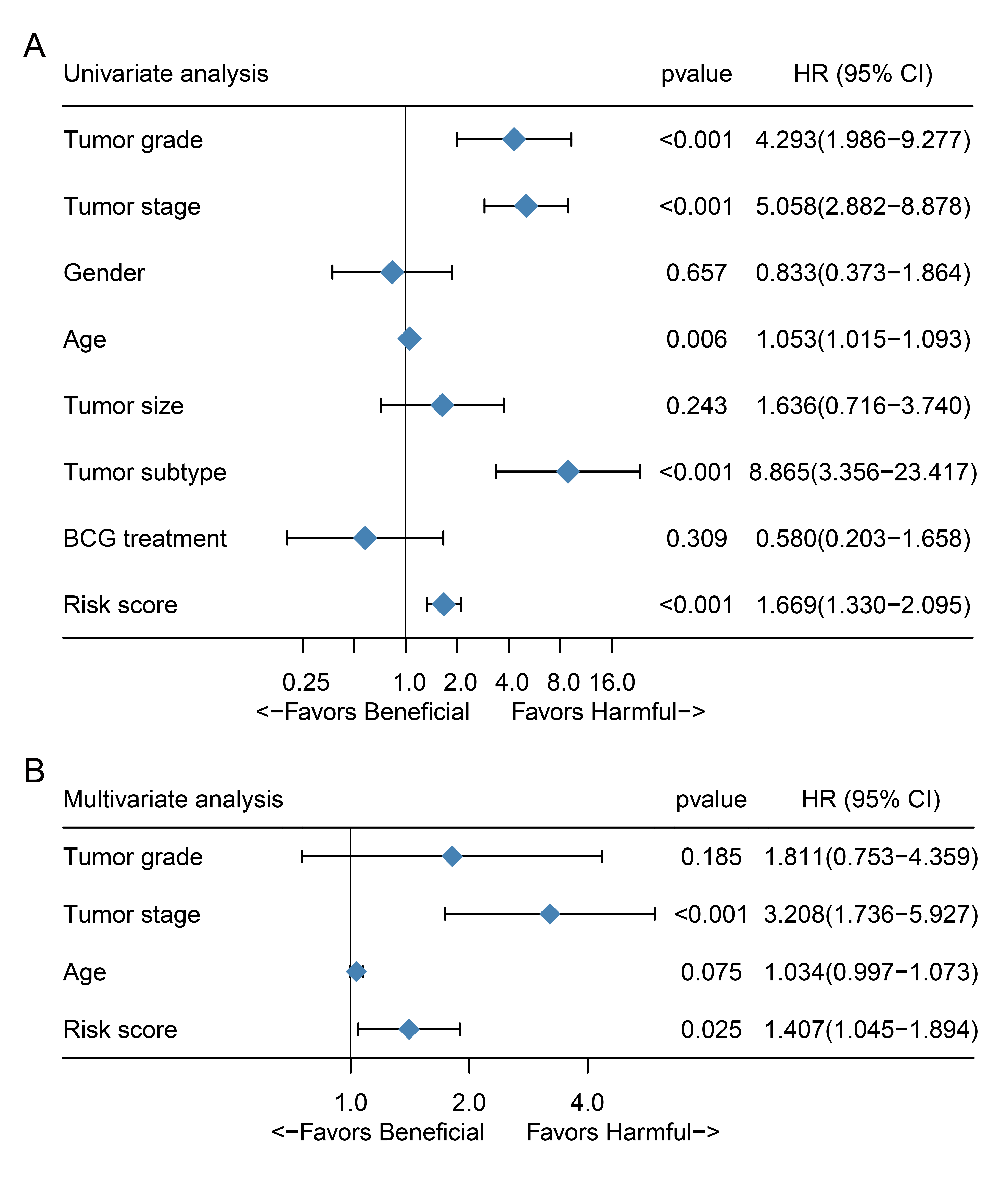


**Supplementary Figure S5. Forest plot summary of univariate and multivariate analysis of outcome model and clinicopathological characters for E-MTAB-4321 dataset.** Univariable (**A**) and multivariable (**B**) analysis of PFS for the BCa patients in E-MTAB-4321 dataset. The blue diamond squares on the transverse lines represent the HR and the gray transverse lines represent 95% CI. And the p value and 95% CI for each clinicopathological character were displayed in detail.


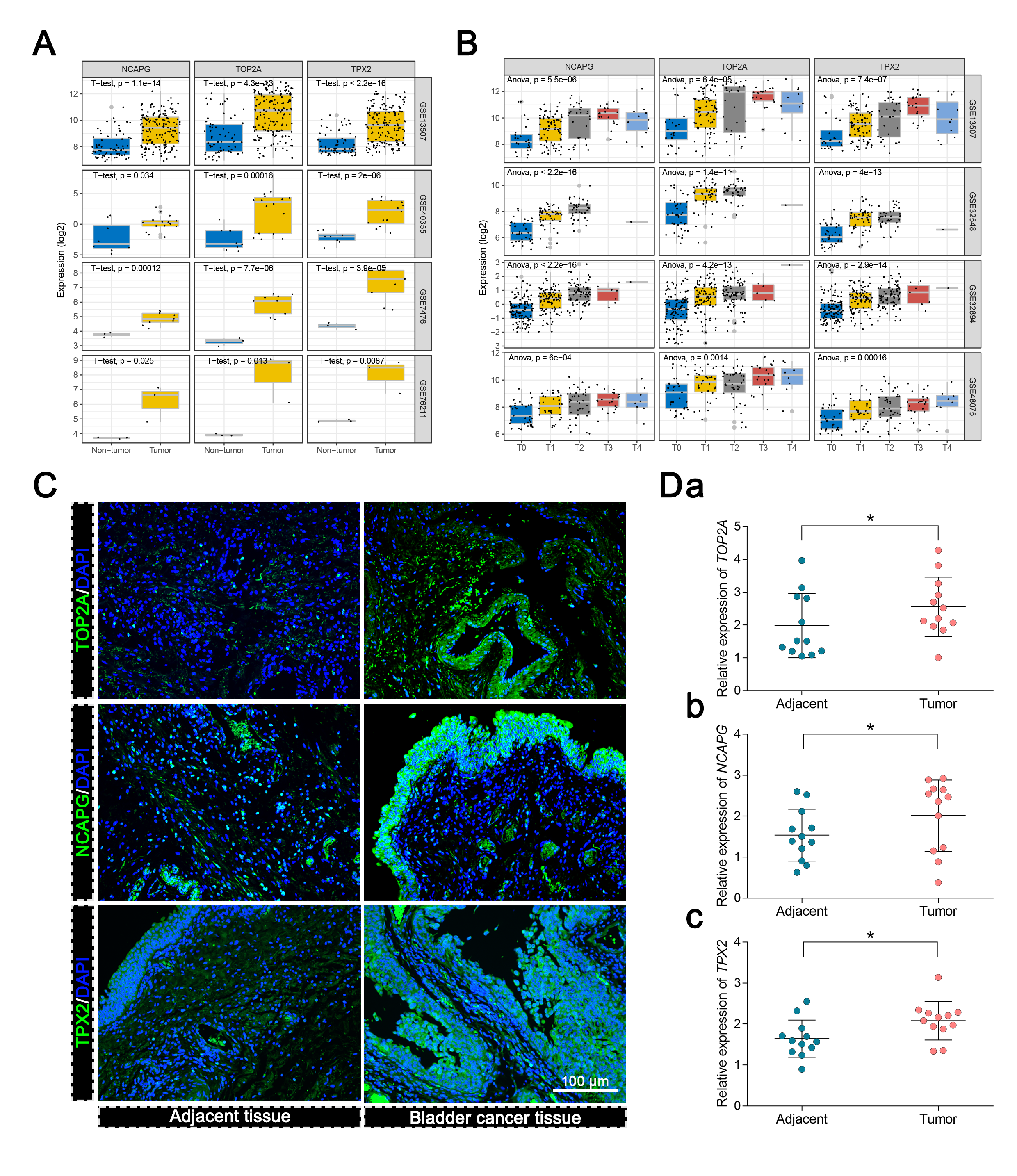


**Supplementary Figure S6. Gene expression validation for 3 candidates.** Expression between tumor and non-tumor based on GSE13507, GSE40355, GSE7476 and GSE76211 datasets (**A**). Expression between different stages of BCa based on GSE13507, GSE32548, GSE32894 and GSE48075 datasets (**B**). Immunofluorescence staining of adjacent tissue and bladder cancer tissue (**C**). QRT-PCR of paired adjacent tissues and bladder cancer tissues (**D**).
