# Supplementary material for: An outcome model for human bladder cancer: A comprehensive study based on weighted gene co‐expression network analysis
Source: J Cell Mol Med. 2019 Dec 28;24(3):2342–55. doi: 10.1111/jcmm.14918 (PMC7011142; doi:10.1111/jcmm.14918)
Supplement: Supplementary file 2 [file JCMM-24-2342-s002.docx]

**Supplementary Table S1. Information of the datasets.**

| GEO  Dataset | Platform | Tumor  type | Total  sample | Non-tumor sample | Tumor sample |
| --- | --- | --- | --- | --- | --- |
| GSE13507 | Illumina human-6 v2.0 expression beadchip | BCa | 256 | 58 | 165 |
|  |  |  |  |  |  |
| GSE32548 | Illumina HumanHT-12 V3.0 expression beadchip | BCa | 131 | 0 | 131 |
|  |  |  |  |  |  |
| GSE32894 | Illumina HumanHT-12 V3.0 expression beadchip | BCa | 308 | 0 | 308 |
|  |  |  |  |  |  |
| GSE48075 | Illumina HumanHT-12 V3.0 expression beadchip | BCa | 142 | 0 | 142 |
|  |  |  |  |  |  |
| GSE7476 | Affymetrix Human Genome U133 Plus 2.0 Array | BCa | 12 | 3 | 9 |
|  |  |  |  |  |  |
| GSE40355 | Agilent-026652 Whole Human Genome Microarray 4x44K v2 (Probe Name version) | BCa | 24 | 8 | 16 |
|  |  |  |  |  |  |
| GSE76211 | Affymetrix Human Transcriptome Array 2.0 [transcript (gene) version] | BCa | 6 | 3 | 3 |
|  |  |  |  |  |  |
| GSE5287 | Affymetrix Human Genome U133A Array | BCa | 30 | 0 | 30 |
|  |  |  |  |  |  |
| E-MTAB-4321 | Illumina HiSeq 2000 | BCa | 462 | 0 | 462 |
