# Supplementary material for: An outcome model for human bladder cancer: A comprehensive study based on weighted gene co‐expression network analysis
Source: J Cell Mol Med. 2019 Dec 28;24(3):2342–55. doi: 10.1111/jcmm.14918 (PMC7011142; doi:10.1111/jcmm.14918)
Supplement: Supplementary file 5 [file JCMM-24-2342-s005.docx]

**Supplementary Table S4. Clinical information of E-MTAB-4321.**

| Clinical information | Patient number |
| --- | --- |
| **Tumor grade** |  |
| PUNLMP | 7 |
| Low grade | 277 |
| High grade | 192 |
| **Disease stage** |  |
| CIS | 3 |
| Ta | 345 |
| T1 | 112 |
| T2-T4 | 16 |
| **Gender** |  |
| Female | 109 |
| Male | 367 |
| **Age** |  |
| Younger (≤65) | 169 |
| Elder (>65) | 307 |
| **Tumor size** |  |
| < 3 | 283 |
| **≥3** | 87 |
| Unknown | 106 |
| **Growth pattern** |  |
| Papillary | 417 |
| Solid | 14 |
| Mixed | 8 |
| Unknown | 37 |
| **BCG treatment** |  |
| Yes | 88 |
| No | 388 |
