# Supplementary material for: An outcome model for human bladder cancer: A comprehensive study based on weighted gene co‐expression network analysis
Source: J Cell Mol Med. 2019 Dec 28;24(3):2342–55. doi: 10.1111/jcmm.14918 (PMC7011142; doi:10.1111/jcmm.14918)
Supplement: Supplementary file 6 [file JCMM-24-2342-s006.docx]

**Supplementary Table S5. List of primary and secondary antibodies**.

| Antigens | Species primary antibodies raised in or species of secondary antibodies’ host | Dilution (IF) | Supplier |
| --- | --- | --- | --- |
| TOP2A | Rabbit, monoclonal | 1:100 | Proteintech, Wuhan, Cat. #20233-1-AP |
| NCAPG | Rabbit, monoclonal | 1:100 | Proteintech, Wuhan, Cat. #11741-1-AP |
| TPX2 | Rabbit, monoclonal | 1:100 | Abclonal, Wuhan, Cat. #11741-1-AP |
| Anti-rabbit IgG (H+L), F (ab') 2 Fragment (Alexa Fluor 555 Conjugate) | Goat | 1:1000 | Boster Biological Technology,  Wuhan, Cat. # BA1105 |
