# Supplementary material for: An outcome model for human bladder cancer: A comprehensive study based on weighted gene co‐expression network analysis
Source: J Cell Mol Med. 2019 Dec 28;24(3):2342–55. doi: 10.1111/jcmm.14918 (PMC7011142; doi:10.1111/jcmm.14918)
Supplement: Supplementary file 7 [file JCMM-24-2342-s007.docx]

**Supplementary Table S6. List of primers for qRT-PCR.**

| Gene symbol | Forward primer | Reverse primer |
| --- | --- | --- |
| TOP2A | ACCATTGCAGCCTGTAAATGA | GGGCGGAGCAAAATATGTTCC |
|  |  |  |
| TPX2 | ATGGAACTGGAGGGCTTTTTC | TGTTGTCAACTGGTTTCAAAGGT |
|  |  |  |
| NCAPG | GAGGCTGCTGTCGATTAAGGA | AACTGTCTTATCATCCATCGTGC |
|  |  |  |
| GAPDH | GGAGCGAGATCCCTCCAAAAT | GGCTGTTGTCATACTTCTCATGG |
